# Renal denervation attenuates cardiac fibrosis and improves left ventricular function in rats with myocardial infarction

**DOI:** 10.1038/s41598-026-50195-w

**Published:** 2026-04-26

**Authors:** Markus Therre, Mathias Hohl, Parisa Aghagolzadeh, Simina-Ramona Selejan, Lucas Lauder, Mert Tokcan, Philipp Markwirth, Harald Engler, Ulrich Hübner, Andreas Müller, Anh Khoa Dennis Huynh, Florian Kahles, Mathias Konstandin, Michael Böhm, Felix Mahfoud

**Affiliations:** 1 Department of Internal Medicine III - Cardiology, Angiology and Intensive Care Medicine, Saarland University Hospital, Homburg, Saar Germany; 2 Faculty of Medicine, Saarland University, Homburg, Saar Germany; 3 Cardiovascular Research Institute Basel (CRIB) and Department of Cardiology, University Heart Center, University Hospital Basel, Basel, Switzerland; 4 Translational Cardiology, Department of Biomedicine, University of Basel and University Hospital Basel, Basel, Switzerland; 5 Institute of Medical Psychology and Behavioral Immunobiology, Center for Translational Neuro- and Behavioral Sciences, University Hospital Essen, Essen, Germany; 6 Department of Clinical Chemistry and Laboratory Medicine, Saarland University Hospital, Homburg, Saar Germany; 7 Department of Diagnostic and Interventional Radiology, Saarland University Hospital, Homburg, Saar Germany; 8 Department of Internal Medicine I - Cardiology, University Hospital Aachen, RWTH Aachen University, Aachen, Germany; 9 Department of Cardiology, Heidelberg University Hospital, Heidelberg, Germany

**Keywords:** Renal denervation, Sympathetic nervous system, Myocardial infarction, Fibrosis, Macrophages, Cardiology, Cardiovascular biology

## Abstract

The sympathetic nervous system (SNS) plays a critical role in regulating inflammatory responses after myocardial infarction (MI). We aimed to investigate whether modulation of the SNS through renal denervation (RDN) influences myocardial remodeling following MI. MI in rats was induced 2 days after bilateral RDN or Sham surgery. Cardiac MRI showed that left ventricular ejection fraction was significantly higher in RDN-treated MI rats compared to Sham-treated MI rats. RNA sequencing of left ventricular tissue revealed that RDN suppresses fibrosis and inflammation related pathways at the whole transcriptome level, with clear separation of the experimental groups. These findings were confirmed by histochemical, gene, and protein expression analyses, which found a significantly higher extent of fibrosis and CD206 expression in the MI rats compared to RDN-treated MI rats. To simulate SNS activity, PMA-differentiated THP-1 cells were incubated with the β-adrenoceptor agonist isoprenaline. This resulted in upregulation of profibrotic macrophage gene expression, which was not the case after co-stimulation with a β1-adrenoceptor antagonist. Modulation of the SNS through RDN mitigates left ventricular remodeling post-MI by reducing myocardial fibrosis and scar size. Heightened SNS activity promotes a profibrotic macrophage phenotype. These findings support investigation into the therapeutic potential of RDN for managing MI patients.

**Supplementary Information:**

The online version contains supplementary material available at 10.1038/s41598-026-50195-w.

## Introduction

Despite advancements in pharmacological therapies and revascularization techniques, myocardial infarction (MI) accompanied by left ventricular (LV) remodeling remains a leading cause of morbidity and mortality globally. For decades, it has been recognized that MI is often complicated by sympathetic nervous system (SNS) activation, which contributes to arrhythmias, vasoconstriction, and scar formation^1–4^. Furthermore, sustained sympathetic activity is a key driver of adverse remodeling after MI, perpetuating a vicious cycle of heart failure (HF) deterioration^5,6^.

The SNS plays a crucial role in regulating immune responses and inflammation^7,8^. Post MI, stress and pain increase bone marrow norepinephrine (NE) and tyrosine hydroxylase – a key enzyme in NE synthesis^9^– levels in mice, which promotes the mobilization of innate immune cells^2,10^. Among these immune cells, macrophages are essential for debris clearing, resolution of inflammation, and wound healing after MI, a process that has been extensively studied in recent years^11–13^. Whole transcriptome analyses in murine models reveal that cardiac macrophages undergo transcriptomic changes throughout the MI timeline: early post-MI macrophages adopt a pro-inflammatory, matrix-degrading phenotype, while macrophages at day 7 exhibit a reparative and fibrogenic phenotype^14^.

Reducing the SNS activity through renal denervation (RDN) presents a novel therapeutic approach to address the deleterious neuroimmune interactions and cardiac remodeling associated with MI. RDN is an interventional approach that modulates efferent and afferent sympathetic nerve fiber activity surrounding the renal arteries, thereby reducing SNS activity. In rat models of experimental MI - whether caused by ischemia/reperfusion injury or permanent left anterior descending (LAD) coronary artery ligation – both surgical and radiofrequency RDN have been shown to improve left ventricular ejection fraction (LVEF)^15–18^. Proposed mechanisms for these beneficial effects include enhanced nitric oxide signaling^16^ and inhibition of renal neprilysin activity^17^. It is also conceivable that RDN may reverse remodeling effects through reduction of the renin-angiotensin-aldosterone system activity^19–21^. Notably, there is emerging evidence suggesting that RDN may attenuate inflammation in ischemia-reperfusion injury^22^. However, the effects of RDN on chronic myocardial fibrosis and cardiac macrophage composition post-MI remain unexplored. This study aimed to determine whether modulation of SNS activity through RDN in a MI rat model would improve cardiac function and remodeling, as well as explore the effects of RDN on cardiac macrophage phenotype. RDN was performed 2 days before LAD ligation to ensure that denervation is established at the time of MI induction.

## Methods

### Animal model

The study was performed in accordance with the German law for the protection of animals and the Directive 2010/63/EU of the European Union. All authors complied with the ARRIVE guidelines. It was approved by the animal ethics committee in Saarbrücken, Germany (#10/2010). 8-week-old male Sprague-Dawley rats were obtained from Charles River (Sulzfeld, Germany) and housed under standardized conditions with access to standard chow 1320 (Altromin, Lage, Germany) and drinking water *ad libitum*. Bilateral chemical and surgical RDN was performed in 8 rats, while 12 sham-operated rats served as controls. After 2 days, sham-operated rats were randomly assigned to either permanent LAD ligation (MI, *n* = 6) or sham surgery (CTRL, *n* = 6). RDN-operated rats were also subjected to LAD ligation (RDN + MI, *n* = 8). Four weeks post MI (terminal timepoint), rats were sacrificed by injection of pentobarbital. Blood samples were obtained during sacrifice by cardiac puncture. Hearts and kidneys were dissected and stored for subsequent analysis. Rat hearts were divided as follows: Tissue from the heart’s midsection (mid-papillary muscle level and comprising the left and right ventricle) was used for histological evaluation and transferred to 4% paraformaldehyde. The remaining lower half of the heart was divided into right ventricle, LV posterior and LV septal-anterior wall. The LV septal-anterior wall was snap frozen in liquid nitrogen for total RNA (RNA bulk sequencing and gene expression analysis via quantitative TaqMan PCR), protein and histological cryo-analysis.

### Bilateral renal denervation

RDN was performed as described previously^23^. Briefly, anesthesia was induced using 1.5–2.5% isoflurane (Piramal Critical Care, Hallbergmoos, Germany). After median laparotomy, both kidneys were surgically denervated by cutting visible nerves and stripping the adventitia from the renal artery, followed by 5 min application of a 20% phenol/ethanol solution with a size 0 brush (Kreul Synthetics, Hallerndorf, Germany) to chemically destroy remaining nerve fibers. In sham-operated animals, the kidneys were exposed without performing RDN.

### Permanent ligation of left anterior descending coronary artery

To achieve experimental MI, permanent ligation of the LAD was carried out as described previously^24^. Rats were anesthetized with 1.5–2.5% isoflurane and a mixture of ketamine/xylazine hydrochloride (80/5 mg/kg i.p.), mechanically ventilated and thoracotomized. LAD was permanently ligated 2 mm distal to aorta, which was verified by blanching of distal epicardium. Sham surgery was identical, however, without LAD occlusion. Post-operative pain management was performed by administering buprenorphine (0.05 mg/kg subcutaneously).

### Magnetic resonance imaging

To assess cardiac function, magnetic resonance imaging (MRI) was performed using a 9.4T animal scanner (Biospec Avance III 94/20, Bruker BioSpin, Germany) under isoflurane anesthesia with continuous electrocardiographic monitoring as described previously^25^. Briefly, cine FLASH sequences were obtained (TR 18ms, TE 1,8ms, FOV 25.6 × 25.6 mm, slice thickness 1 mm, interslice gap 0.5 mm) and six to eight two-chamber sequences of the left ventricle (LV) were analyzed with Segment v2.0 R4265 (Medviso, Sweden) to determine endsystolic (ESV) and enddiastolic volumes (EDV). LVEF was calculated by dividing stroke volume (difference between EDV and ESV) by EDV. Additionally, peak filling rate (PFR) and peak ejection rate (PER) of the LV were determined.

### Determination of renal norepinephrine content

Snap frozen kidney tissue was minced in TRIS-EDTA and centrifugated. Norepinephrine (NE) content of the supernatant was analyzed by high pressure liquid chromatography (HPLC) using Chromsystems plasma catecholamines kit (Chromsystems Instruments & Chemicals GmbH, Gräfelfing, Germany) according to the manufacturer’s protocol.

### Determination of plasma concentrations of kidney and liver function parameters, electrolytes, and glucose

Plasma concentrations of kidney and liver function parameters, glucose and calcium were analyzed after final blood sampling on the Cobas 8000 (c702 module, Roche Diagnostics GmbH, Mannheim, Germany) automatic chemistry analyzer using the manufacturer’s reagents, calibrators and quality controls. The methods were as follows: creatinine (colorimetric assay based on the Jaffé method), urea (enzymatic assay with urease and glutamate dehydrogenase), AST and ALT (assays according to the International Federation of Clinical Chemistry (IFCC) with pyridoxal phosphate activation), albumine (colorimetric assay with bromcresol green), glucose (enzymatic reference method with hexokinase) and calcium (photometric assay with the chromophore 5-nitro-5′-methyl-(1,2-bis(o-aminophenoxy)ethan-N, N,N′,N′-tetraacetic acid (NM-BAPTA)). Plasma concentrations of sodium and potassium were measured using the indirect ion-selective electrode (ISE) method (Cobas 8000, ISE analytical unit, Roche Diagnostics).

### Total RNA isolation and gene expression analysis

RNA was extracted from snap frozen LV septal-anterior wall tissue using peqGold TriFast (PeqLab, Erlangen, Germany) reagent following the manufacturer’s protocol. Two µg of RNA were digested with DNAse (Peqlab, Erlangen, Germany) and reverse transcribed using the HighCap cDNA RT Kit (Applied Biosystems, Darmstadt, Germany). TaqMan PCR was conducted in a StepOne plus thermocycler (Applied Biosystems, Darmstadt, Germany) using TaqMan GenEx Mastermix (Applied Biosystems, Darmstadt, Germany). Glyceraldehyde-3-phosphate dehydrogenase (GAPDH) was taken as a housekeeping gene to normalize gene expression. The ΔCt was used for statistical analysis and 2 − ΔΔCt for data presentation. TaqMan probes (all Applied Biosystems, Darmstadt, Germany) can be found in the Supplemental material online.

### RNA bulk sequencing and transcriptomic analysis

Total RNA (100 ng) was used for RNA-seq library preparation using the TruSeq Stranded Total RNA Library Prep Kit with Ribo-Zero Gold (Illumina), following the manufacturer’s instructions. For final library amplification, TruSeq amplification reagents were replaced with the Equinox Library Amplification Kit (Watchmaker Genomics) using the supplied P5/P7 primer mix and 12 PCR cycles. Paired-end sequencing (2 × 61 cycles) was performed on a NovaSeq 6000 at the Genomics Facility Basel according to the manufacturer’s guidelines. For downstream analysis, processed expression data were analyzed in Qlucore Omics Explorer (v3.10) to generate the PCA, differential expression analyses (volcano plots) and Venn diagram. Differentially expressed genes were defined using an absolute fold-change threshold of ≥ 2.5 together with a significance threshold of *P* < 0.05; where multiple-testing correction was applied, an FDR-adjusted P value < 0.1 was used. Gene set enrichment analysis was performed using the GSEA desktop application, and enrichment plots were reported with normalized enrichment scores (NES) and P values.

### Western blot analysis

Kidney and LV tissue was minced in homogenization buffer with complete protease inhibitor (Roche, Penzberg, Germany) and PMSF (1 mmol/L) and centrifuged. Fifty µg of protein were separated with SDS-PAGE (10%) and transferred to nitrocellulose membranes (Bio-Rad, Feldkirchen, Germany). Membranes were blocked in phosphate-buffered saline (PBS) containing 0.1% Tween and 5% non-fat dry milk for 120 min at room temperature (RT) and exposed to primary antibodies. The next day, respective secondary antibodies were incubated for 60 min at RT and analyzed by enhanced chemi-luminescence (Amersham Pharmacia Biotech, Amersham, UK) using the Fusion SL gel documentation system (Peqlab, Erlangen, Germany). Data are presented as integral optical density (IOD) normalized to GAPDH and all figures contain representative blots. Antibodies and dilutions as well as full-length uncropped gels can be found in the Supplemental material online. Blot presentations comply with the digital image and integrity policies of *Scientific Reports.* Blots show representative individual responses and were cropped to improve clarity and conciseness of the presentation, which was made explicit by clear delineation with white space.

### Histology and immunofluorescence staining

Whole heart tissue (midsection as described above) was fixed in 4% paraformaldehyde for 48 h and imbedded in paraffin. Tissue sections of 3 μm were fixed at 56 °C overnight, deparaffinized, rehydrated and stained with picrosirius red (Morphisto, Frankfurt am Main, Germany) to visualize the amount of fibrosis. An Aperio ImageScope x64 whole slide scanner (Leica, Wetzlar, Germany) was used for image acquisition which was additionally performed by polarized microscopy for distribution of collagen type I (red-yellow birefringence) and collagen type III (green birefringence). For analysis, NIS-Elements BR 3.2 (Nikon, Melville, USA) was used, and scar size was calculated as the ratio of picrosirius red positively stained area over total area (at the midpapillary level). For immunofluorescence staining of CD206 in LV anterior-septal wall sections snap-frozen tissue was embedded in tissue freezing medium (Leica, Wetzlar, Germany) and cryosectioned to slices of 6 μm. After washing in PBS and blocking in PBS with 10% donkey serum slices were incubated with primary antibody at 4 °C in a moisture chamber overnight. As a negative control, rabbit serum (the host species of the Anti-CD206 antibody), diluted to the same concentration as the primary antibody was used. To remove unbound antibodies, slices were washed twice in PBS followed by incubation with secondary antibody at 37 °C for 2 h. Antibodies and respective dilutions can be found in the Supplemental material online. Finally, slides were mounted with mounting medium containing DAPI (Vector Laboratories, Burlingame, USA) and images were acquired as described above. Sections were collected at comparable LV levels across animals. Fields of view were selected at the whole LV (in congruence to gene and protein expression analyses) except for the scar area which was excluded. CD206 stained cells were quantified per field of view (FOV) in 40x magnification. Only clearly identifiable single cells, each with a DAPI-positive stained nucleus, were included in the analysis.

### THP-1 cell culture

The human monocytic cell line THP-1 was purchased from ATCC (Wesel, Germany). Briefly, 500,000 cells were distributed to uncoated 12-well dishes. Each well was covered with 1 ml RPMI 1640 medium (Thermo Fisher Scientific, Waltham, USA) containing 10% fetal bovine serum and 1% penicillin/streptomycine and cells were incubated at 37 °C and 5% CO2. To induce differentiation to macrophages, cells were incubated with 50 nM phorbol 12-myristate 13-acetate (PMA) for 24 h. Stimulation experiments were performed with β1-adrenoceptor agonist isoprenaline (ISO, 0.1 µmol/L) in the presence or absence of the respective β-adrenoceptor antagonists CGP 201,712 A (β1-selective, 0.3 µmol/L) and ICI 118.551 (β2-selective, 0.1 µmol/L) over a period of 72 h with daily stimulations. After 72 h, cells were harvested and processed for gene expression analysis as described above. Data are obtained as duplicates from at least three independent experiments.

### Statistics

Statistical analysis was conducted with GraphPad Prism 10 (GraphPad Software, Boston, MA, USA). Data are presented as mean ± SEM if not otherwise specified and were tested for normal distribution using the Shapiro-Wilk or D’Agostino & Pearson normality test. If normality was assumed, between-group differences were assessed using univariate analysis of variance (ANOVA) with Tukey post-hoc test. The non-parametric Kruskal-Wallis test was used for non-normally distributed variables or when the sample size was < 6. For comparison of normally distributed variables between 2 groups, an unpaired, 2-tailed t-test was applied. A 2-sided p-value < 0.05 was considered statistically significant.

## Results

### Effective RDN mitigates systolic LV dysfunction in rats with MI

**Fig. 1:**
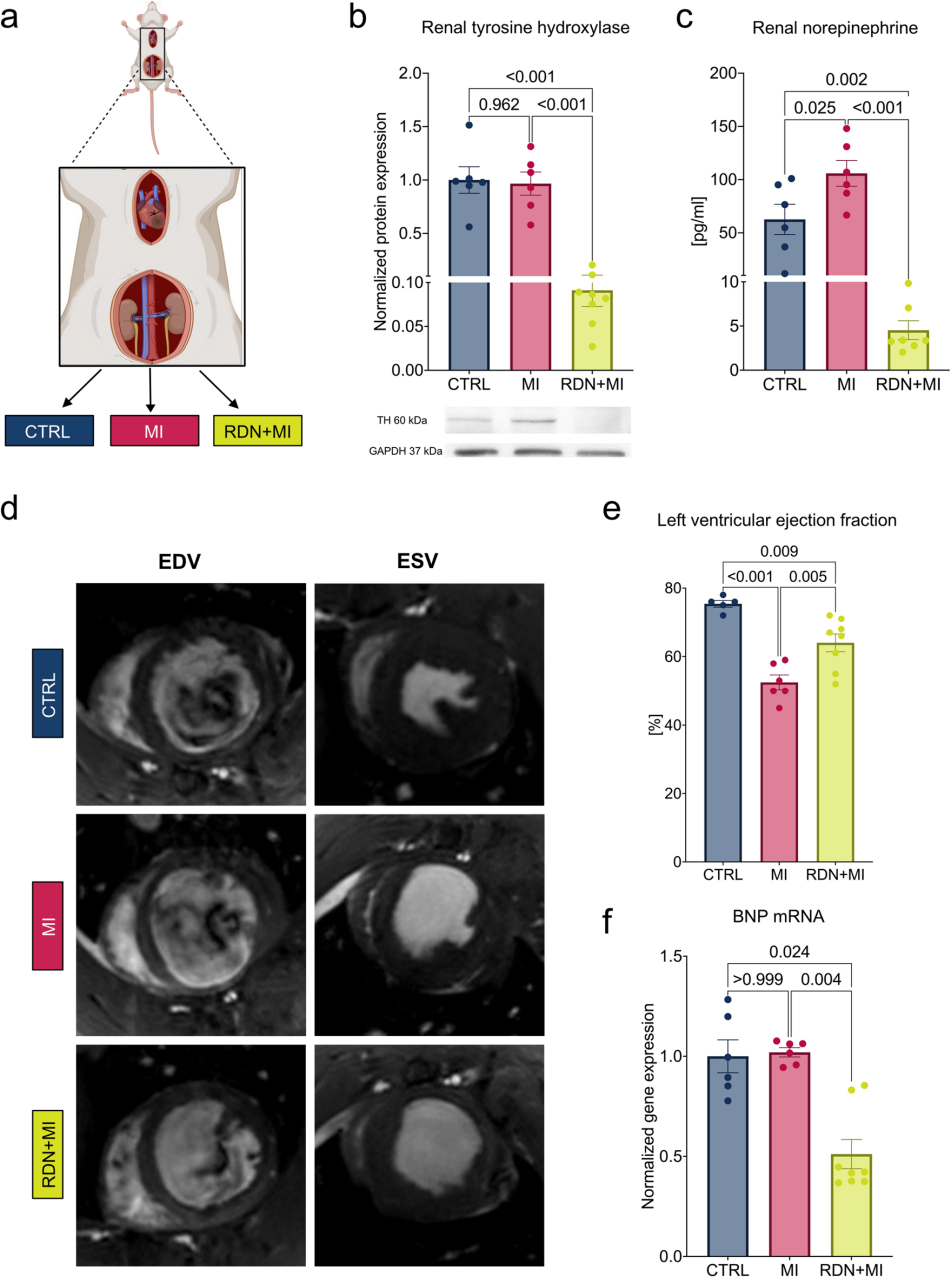
Effective RDN mitigates systolic LV dysfunction in rats with MI. (**a**) Experimental setup: RDN was performed in 8 rats, while 12 sham-operated rats served as controls. After 2 days, sham-operated rats were randomly assigned to either permanent LAD ligation (MI, n=6) or sham-sham surgery (CTRL, n=6). RDN-operated rats were also subjected to LAD ligation (RDN+MI, n=8). After 4 weeks, all animals were sacrificed for biochemical and histological analysis. (**b**) Protein expression analysis revealed significant less TH in RDN+MI (n=8) compared with CTRL (n=6) or MI (n=6). Blots show representative individual responses and were cropped to improve clarity and conciseness of the presentation. Original gels are presented in Supplemental material online. (**c**) HPLC demonstrated significant higher renal norepinephrine (NE) concentrations after MI (n=6) compared to CTRL (n=6), which were significantly smaller in RDN+MI (n=7). (**d**) Representative cardiac MRIs depicting two-chamber sequences of LV enddiastolic (EDV) and endsystolic (ESV) volumina in CTRL, MI and RDN+MI. (**e**) MI rats (n=6) displayed reduced LVEF when compared with CTRL (n=5), which was higher in RDN+MI (n=8). (**f**) Gene expression analysis revealed significant attenuation of myocardial BNP in RDN+MI (n=8) compared with CTRL (n=6) or MI (n=6). Data are shown as mean±SEM. P-value was determined using ANOVA with Tukey’s test for multiple comparisons or using Kruskal-Wallis test if normality could not be assumed.CTRL=Control group (Sham RDN-Sham MI); MI=Myocardial infarction group (Sham RDN-MI); RDN+MI=Renal Denervation and myocardial infarction group (RDN-MI); TH=Tyrosine hydroxylase; GAPDH=glyceraldehyde-3-phosphate dehydrogenase; BNP=Brain natriuretic peptide.

A total of 20 8-week-old Sprague-Dawley rats were randomly assigned to undergo RDN and LAD ligation (RDN + MI, *n* = 8), sham-RDN and LAD ligation (MI, *n* = 6), or sham-RDN and sham-LAD ligation (CTRL, *n* = 6). RDN or sham-RDN was performed 2 days prior to permanent LAD ligation or sham-LAD ligation (Fig. 1a). After four weeks, protein expression analysis of the kidneys confirmed the effectiveness of RDN, with a significant 90.9% downregulation of the enzyme tyrosine hydroxylase (TH) in RDN + MI when compared with CTRL (*p* < 0.001) (Fig. 1b). Renal NE concentrations were nearly double in the MI group compared to CTRL (105.9 ± 12.1 vs. 62.7 ± 14.2 pg/ml, *p* = 0.025) (Fig. 1c). In contrast, renal NE levels were significantly smaller in RDN + MI (4.5 ± 1.1 pg/ml, *p* < 0.001 vs. MI) (Fig. 1c), demonstrating the sympatholytic effect of RDN. Clinical laboratory parameters, including kidney and liver function parameters, were not significantly altered in the MI or RDN + MI groups compared to CTRL (Table 1). Cardiac function was assessed using MRI scans conducted 4 weeks after MI. Figure 1d depicts representative cardiac MRIs images of two-chamber sequences of LV enddiastolic (EDV) and endsystolic (ESV) volumina in CTRL (*n* = 5), MI (*n* = 6) and RDN + MI (*n* = 8) animals. LVEF was significantly reduced in the MI group compared to CTRL (52.5 ± 2.2 vs. 75.4 ± 1.0%, *p* < 0.001) (Fig. 1e). However, LVEF was significantly higher in the RDN + MI group (64.0 ± 2.6%, *p* = 0.005 vs. MI) (Fig. 1e). Quantification of EDV, ESV, SV, PFR and PER can be found in the Supplemental material online. In line with these functional improvements, myocardial BNP gene expression was 48.9 ± 7.3% smaller in RDN + MI when compared to MI (*p* = 0.004) (Fig. 1f).

**Table 1:** Clinical laboratory parameters of CTRL, MI and RDN + MI rats 4 weeks after MI.

Parameter (unit)	CTRL Mean (SEM)	MI Mean (SEM)	RDN + MI Mean (SEM)	*p*-values		
				CTRL vs. MI	CTRL vs. RDN + MI	MI vs. RDN + MI
Creatinine (mg/dL)	0.33 (0.02)	0.38 (0.02)	0.35 (0.02)	0.161	0.766	0.414
Urea (mg/dL)	36.40 (1.29)	40.50 (4.63)	35.50 (1.78)	0.642	0.978	0.492
Albumine (g/L)	37.40 (0.40)	38.00 (1.16)	36.17 (1.47)	0.933	0.748	0.505
Potassium (mmol/L)	5.98 (0.32)	5.85 (0.40)	6.60 (0.31)	0.964	0.455	0.294
Sodium (mmol/L)	141.40 (0.93)	142.83 (0.70)	143.33 (0.92)	0.490	0.287	0.905
Calcium (mmol/L)	2.50 (0.05)	2.68 (0.05)	2.63 (0.06)	0.103	0.274	0.804
Glucose (mg/dL)	216.20 (15.82)	285.50 (24.56)	286.50 (33.03)	0.202	0.193	0.999
AST (U/L)	158.40 (7.34)	141.17 (15.37)	154.33 (15.14)	0.668	0.977	0.770
ALT (U/L)	63.80 (4.54)	58.50 (4.65)	57.67 (2.89)	0.647	0.562	0.988

CTRL=Control group (Sham RDN-Sham MI); MI=Myocardial infarction group (Sham RDN-MI); RDN + MI=Renal Denervation and myocardial infarction group (RDN-MI); AST=aspartate transaminase; ALT=alanine transaminase; SEM=standard error of the mean.

### Bulk RNA-seq identifies ECM remodeling and inflammatory programs associated with RDN cardioprotection in rats with MI

**Fig. 2:**
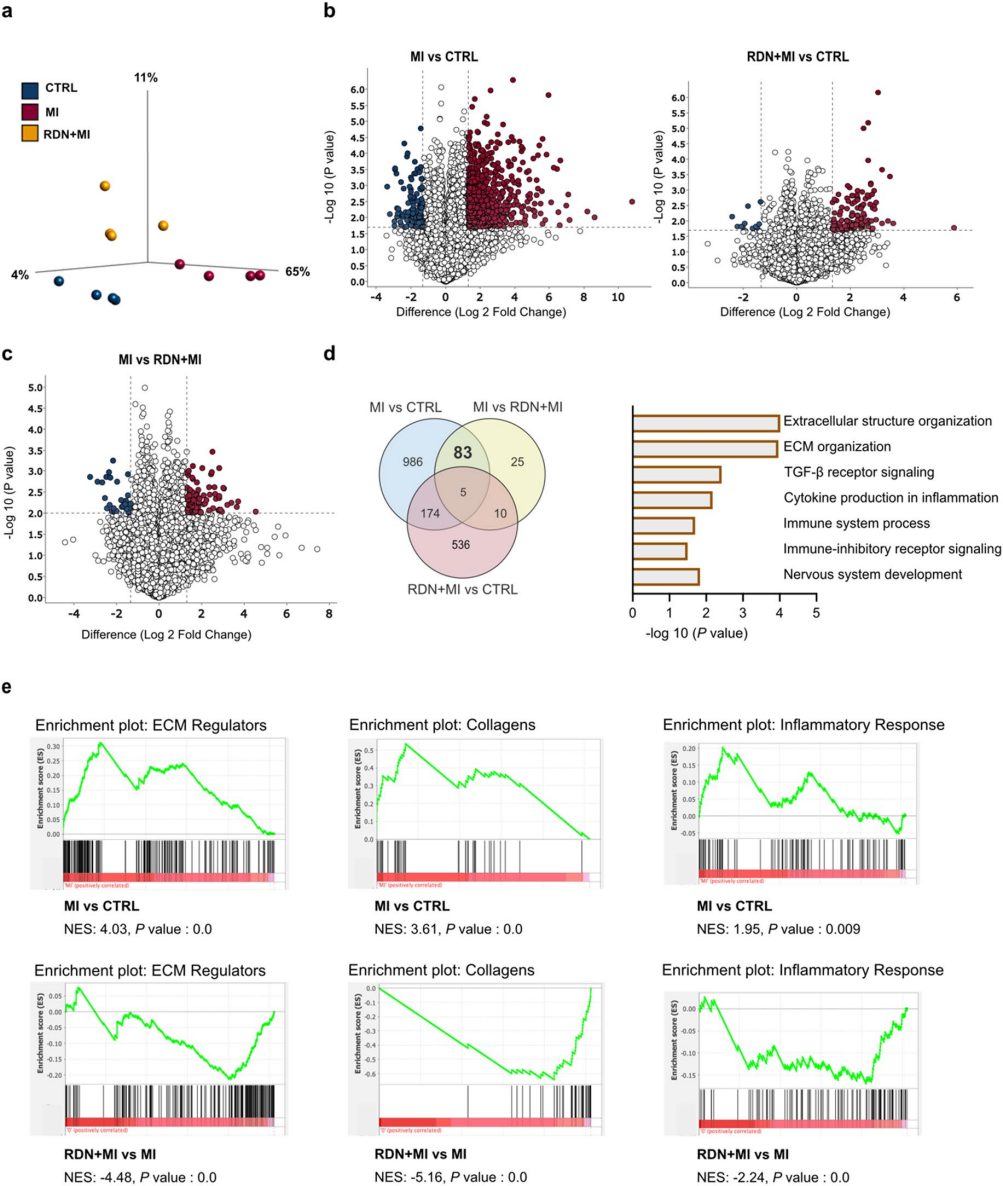
Bulk RNA-seq identifies ECM remodeling and inflammatory programs associated with RDN cardioprotection in rats with MI. Bulk RNA-sequencing was performed on LV septal-anterior tissue collected at study end from CTRL, MI, and RDN + MI groups (*n* = 4 per group). (**a**) Principal component analysis (PCA) of LV transcriptomes showing separation of MI from CTRL and a shift of RDN + MI away from MI. (**b**) Volcano plots showing differential gene expression for MI vs. CTRL (*n* = 1,248 DE genes) and RDN + MI vs. CTRL (*n* = 725 DE genes). (**c**) Volcano plot comparing MI vs. RDN + MI (*n* = 123 DE genes), highlighting transcriptional differences between infarcted hearts with and without RDN. (**d**) Venn diagram showing overlap of differentially expressed genes across comparisons. The 83 gene subset represents genes significant in MI vs. CTRL and MI vs. RDN + MI, but not significant in RDN + MI vs. CTRL (MI associated changes no longer detectable after RDN). Right: Gene ontology enrichment analysis of the 83 genes (bar plot shows -log10 P value). (**e**) Gene set enrichment analysis (GSEA) enrichment plots for representative pathways (ECM Regulators, Collagens, Inflammatory Response) in MI vs. CTRL (top) and RDN + MI vs. MI (bottom). Normalized enrichment scores (NES) and nominal P values are shown in the plots. CTRL=Control group (Sham RDN-Sham MI); MI=Myocardial infarction group (Sham RDN-MI); RDN + MI=Renal Denervation and myocardial infarction group (RDN-MI); BNP=Brain natriuretic peptide.

To investigate the molecular mechanisms underlying the cardioprotective effects of RDN in MI, bulk RNA-sequencing on LV septal-anterior tissue were performed (*n* = 4 per group). Unsupervised dimensionality reduction (PCA) demonstrated clear separation of MI from CTRL, with the RDN + MI group shifting away from MI (and toward CTRL), indicating a RDN-associated modulation of the post-MI transcriptomic response (Fig. 2a). Consistent with this global pattern, differential expression analysis identified 1,248 differentially expressed genes in MI vs. CTRL, whereas RDN + MI vs. CTRL showed 725 differentially expressed genes (Fig. 2b). Direct comparison of MI vs. RDN + MI identified 123 differentially expressed genes, reflecting transcriptional differences between infarcted hearts with and without RDN (Fig. 2c). A subset of 83 genes was defined by the following pattern: they were significantly altered in MI vs. CTRL and also significantly different in MI vs. RDN + MI, but not significantly different in RDN + MI vs. CTRL. This gene set therefore captures MI-associated transcriptional changes that are no longer detectable upon RDN treatment, consistent with prevention of MI-driven dysregulation by RDN (Fig. 2d). Gene ontology enrichment analysis of these 83 genes highlighted biological processes central to adverse post-MI remodeling, including ECM organization/extracellular structure organization (fibrosis related remodeling), TGF-β receptor signaling and immune related processes linked to inflammation and immune system regulation (Fig. 2e). In line with these findings, gene set enrichment analysis (GSEA) demonstrated enrichment of ECM regulators, collagen associated programs, and inflammatory response in MI vs. CTRL, whereas these pathways were significantly attenuated in RDN + MI vs. MI (Fig. 2e). Together, the RNA-seq data suggest that RDN modulates post-MI remodeling at the molecular level by reducing inflammatory and ECM/fibrosis-associated transcriptional programs, which were validated using tissue level scar/fibrosis quantification in the subsequent analyses.

### RDN-treated rats exhibit smaller MI scar sizes and less myocardial fibrosis

**Fig. 3:**
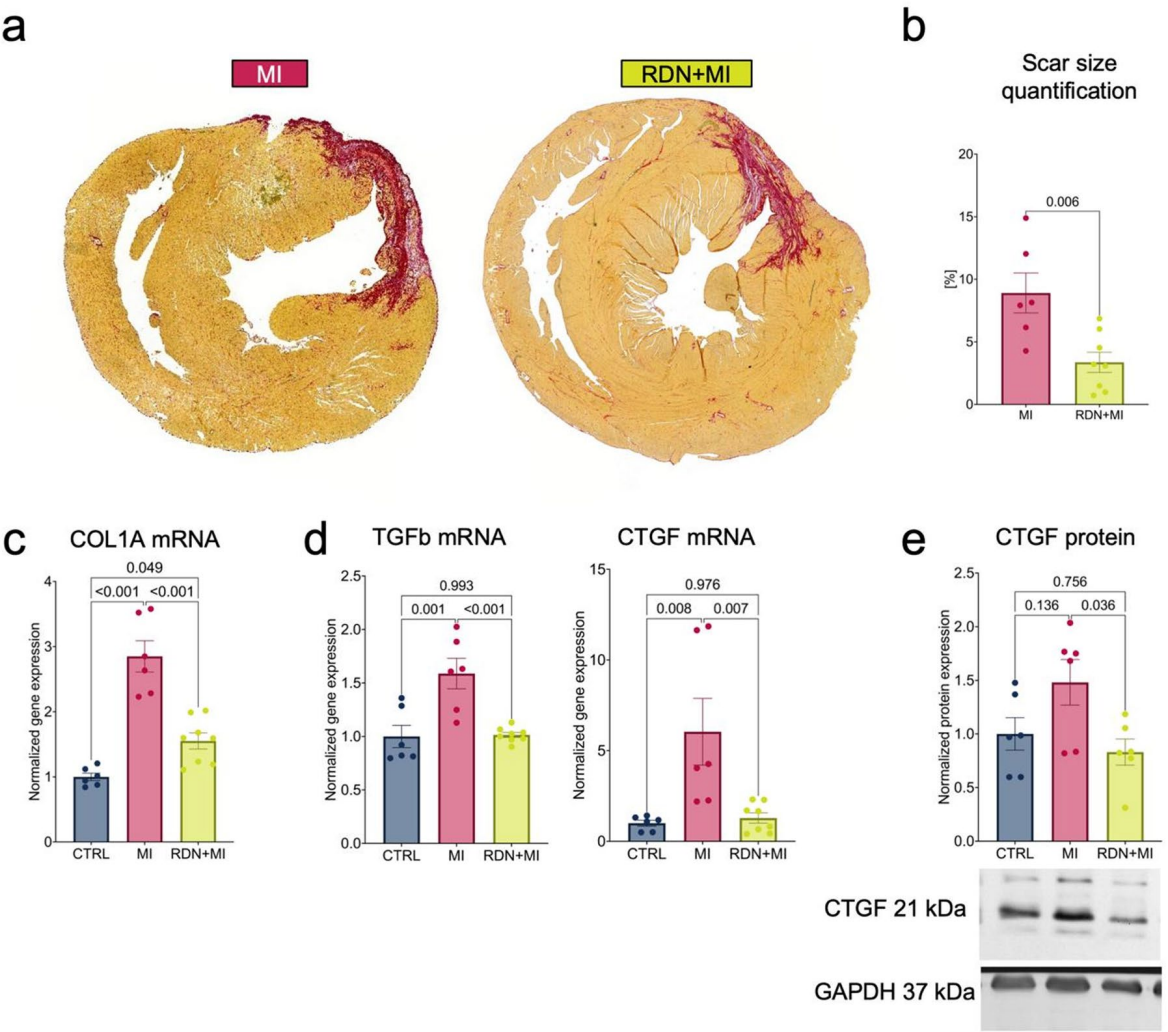
RDN-treated rats exhibit smaller MI scar sizes and less myocardial fibrosis. (**a**) Representative images of Sirius Red-stained hearts of MI and RDN+MI rats. Collagen of the MI scar appears red on a yellow background. (**b**) Quantification of MI scar size (defined as percent area of fibrotic scar within the area of the LV) revealed significant smaller scars in RDN+MI (n=8) compared to MI (n=6) rats. (**c**) Myocardium of the LV anterior -septal wall was analyzed for fibrogenic genes and proteins. COL1A gene expression was higher in MI (n=6) compared to CTRL (n=6) and smaller in RDN+MI (n=8). (**d**) CTGF and TGFb gene expression was higher in MI (n=6) compared to CTRL (n=6) and smaller in RDN+MI (n=8). (**e**) C-terminal CTGF protein expression was also higher in MI (n=6) compared to CTRL (n=6) but smaller in RDN+MI (n=6). Blots show representative individual responses and were cropped to improve clarity and conciseness of the presentation. Original gels are presented in Supplemental material online. Data are shown as mean ± SEM. P-value was determined using ANOVA with Tukey’s test for multiple comparisons. An unpaired t-test was used for comparison of two groups.CTRL=Control group (Sham RDN-Sham MI); MI=Myocardial infarction group (Sham RDN-MI); RDN+MI=Renal Denervation and myocardial infarction group (RDN-MI); COL1A=Type I collagen A; CTGF=Connective tissue growth factor; TGFb=transforming growth factor beta 1; GAPDH=glyceraldehyde-3-phosphate dehydrogenase; mRNA=messenger ribonucleic acid.

Four weeks after LAD ligation or sham-LAD ligation, the animals were sacrificed, and the hearts were histochemically stained with Sirius Red to visualize collagen of the myocardial scar (Fig. 3a). Scar sizes were significantly smaller in RDN + MI than MI (3.4 ± 0.8% vs. 8.9 ± 1.6, *p* = 0.006, Fig. 3b). Gene expression of type I collagen (COL1A, 185.0 ± 24.0%, *p* < 0.001 vs. CTRL), TGFb (58.8 ± 14.2%, *p* = 0.001), and CTGF (504.2 ± 183.9%, *p* = 0.008), which are markers for fibrogenic remodeling, were significantly smaller in LV myocardium in the RDN + MI group (COL1A *p* < 0.001 vs. MI, TGFb *p* < 0.001, CTGF *p* = 0.007, Fig. 3c and d). CTGF protein expression was significantly reduced in the RDN + MI group (*p* = 0.036 vs. MI, Fig. 3e).

### Post-ischemic fibrosis is associated with CD206 expression which is reduced in RDN-treated rats

**Fig. 4:**
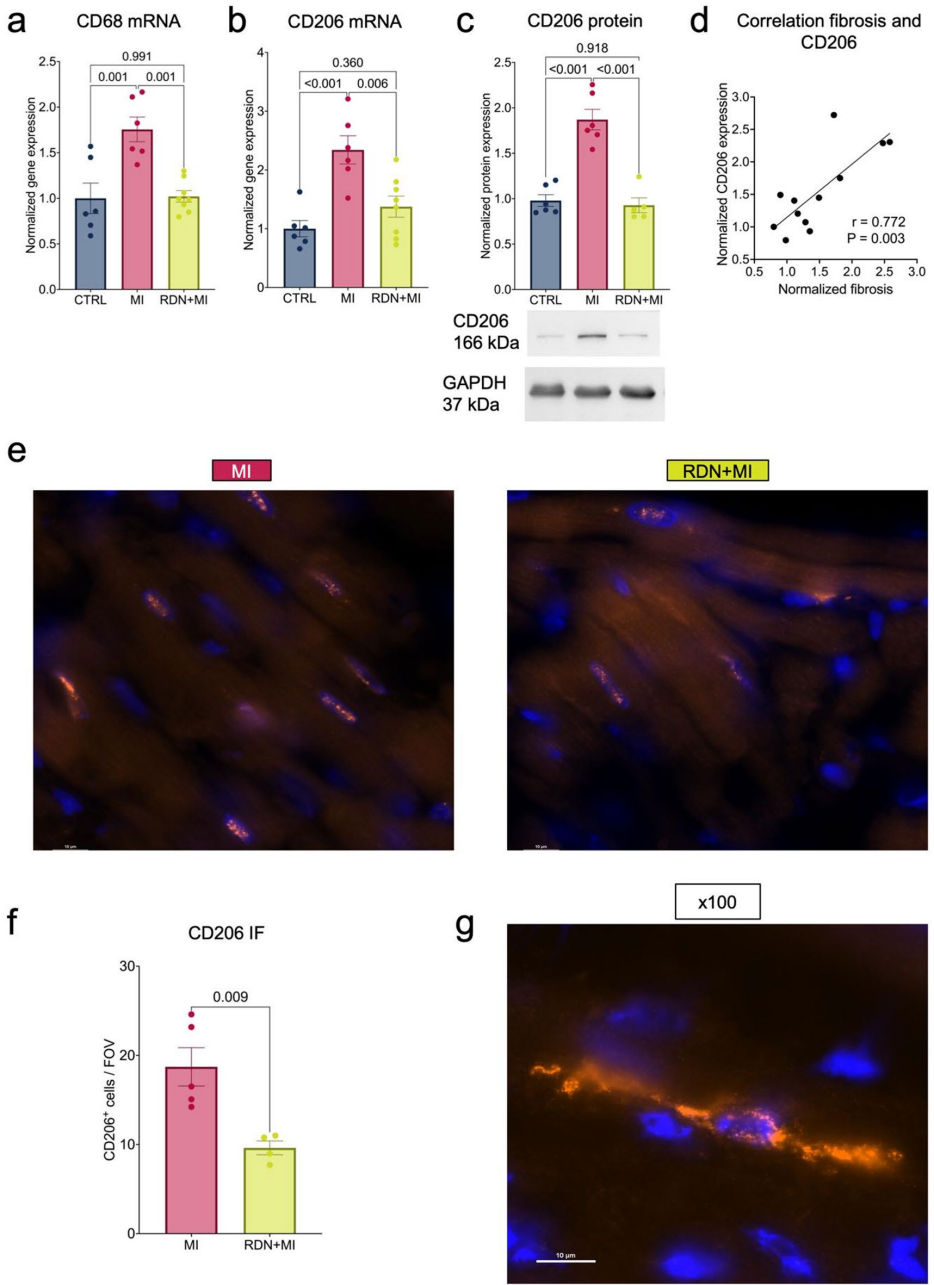
Post-ischemic fibrosis is associated with CD206-expressing macrophages which are reduced in RDN-treated rats. (**a**) LV septal-anterior tissue was analyzed for macrophage phenotype markers. CD68 gene expression was elevated after MI (n=6) compared to CTRL (n=6), which was reversed in RDN+MI (n=8). (**b**) CD206 gene expression as marker for a profibrotic macrophage phenotype was higher in MI (n=6) compared to CTRL (n=6) but smaller in RDN+MI (n=8) compared to MI. (**c**) CD206 protein expression was also elevated in MI (n=6) compared to CTRL (n=6) but not in RDN+MI (n=5) compared to CTRL. Blots show representative individual responses and were cropped to improve clarity and conciseness of the presentation. Original gels are presented in Supplemental material online. (**d**) Gene and protein expression of above mentioned fibrogenic markers as well as of CD206 were averaged and normalized. Pearson correlation revealed significant correlation of CD206 with fibrosis (n=12). (**e**) Representative immunofluorescence images (x63 magnification) of CD206-stained hearts of MI and RDN+MI rats. CD206 appears orange on a black background, while nuclei are blue. (**f**) Quantification of number of CD206-expressing macrophages per field of view revealed significant smaller number in RDN+MI (n=4) compared to MI (n=5) rats. (g) Representative immunofluorescence image (x100 magnification) of CD206-stained macrophage of the heart of a MI rat reveals the characteristic spindle-like shape. Data are shown as mean ± SEM. P-value was determined using ANOVA with Tukey’s test for multiple comparisons. An unpaired t-test was used for comparison of two groups.CTRL=Control group (Sham RDN-Sham MI); MI=Myocardial infarction group (Sham RDN-MI); RDN+MI=Renal Denervation and myocardial infarction group (RDN-MI); CD=cluster of differentiation; GAPDH=glyceraldehyde-3-phosphate dehydrogenase; mRNA=messenger ribonucleic acid; FOV=field of view; IF=immunofluorescence.

Analysis of LV anterior-septal myocardium revealed elevated gene expression of CD68, a marker for macrophages, by 75.5 ± 13.6% (*p* = 0.002 vs. CTRL) in MI, but this elevation was not observed in RDN + MI (*p* = 0.991) (Fig. 4a). CD68 gene expression was significantly lower in the RDN + MI group compared to MI (*p* = 0.001). The analysis of gene and protein expression of CD206, a marker for reparative and fibrogenic macrophages, was 134.2 ± 24.0% and 87.0 ± 11.4% higher in the MI group (*p* = 0.001 and *p* < 0.001 vs. CTRL). In RDN + MI, CD206 expression was not different to CTRL levels but significantly smaller compared to MI (*p* = 0.006 and *p* < 0.001 vs. MI, Fig. 4b and c). Immunofluorescence staining confirmed fewer CD206-expressing macrophages per field of view in RDN + MI (9.6 ± 0.8 CD206^+^ cells) compared to MI (18.7 ± 2.2 CD206^+^ cells, *p* = 0.009, Fig. 4f). A positive correlation between CD206-expressing macrophages and myocardial fibrosis was observed (*r* = 0.772, *P* = 0.003, Fig. 4d). Interestingly, CD206-expressing macrophages typically exhibited a spindle-shaped morphology (Fig. 4g). Gene expression of CXC motif chemokine ligand 10 (CXCL10), a marker for proinflammatory macrophages, was inversely proportional to CD206 and 63.0 ± 4.6% smaller in MI compared to CTRL (Supplemental material online). Multiplex cytokine analysis of serum samples 4 weeks post-MI revealed no significant differences between the 3 groups (Supplemental material online).

### β-adrenergic stimulation of macrophages causes upregulation of profibrotic and downregulation of inflammatory genes via β1-adrenoceptor

**Fig. 5:**
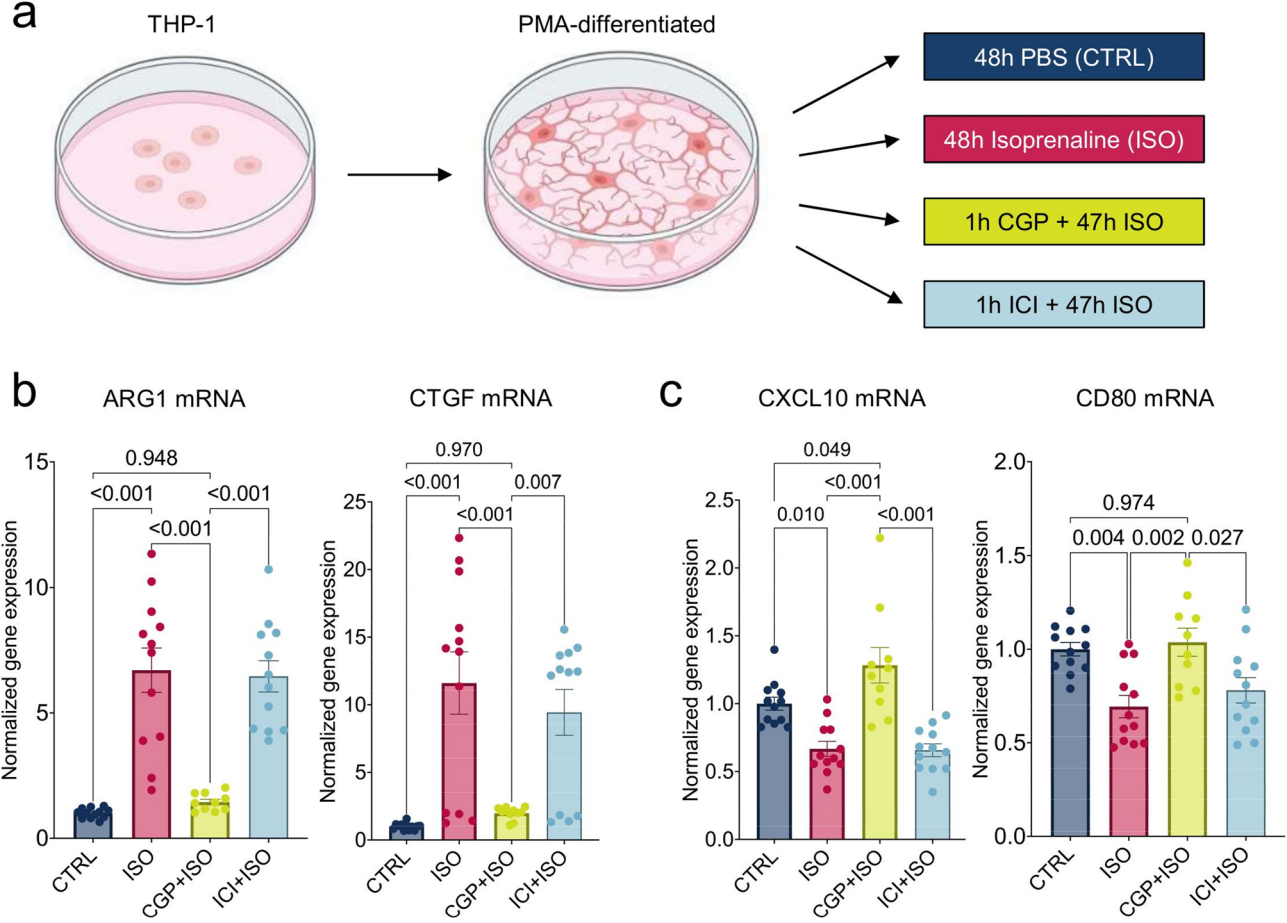
β-adrenergic stimulation of macrophages causes upregulation of profibrotic and downregulation of inflammatory genes via β1-adrenoceptor. (**a**) Experimental setup: THP-1 cells were differentiated to macrophages by stimulation with 50 nM PMA for 24 h, followed by stimulation with β-adrenoceptor agonist ISO or PBS (CTRL) in the presence or absence of β1-adrenoceptor antagonist CGP or β2-adrenoceptor antagonist ICI over a period of 72 h with daily stimulations. Cells were harvested and gene expression analysis was conducted for macrophage phenotype markers. (**b**) Arginase 1 (ARG1) and CTGF genes were higher in ISO compared to CTRL. Compared to ISO, expression was smaller in CGP + ISO but not ICI + ISO. (**c**) CXC motif chemokine ligand 10 (CXCL10) and CD80 gene expression were significantly smaller in ISO compared to CTRL. Compared to ISO, expression was higher in CGP + ISO but not ICI + ISO. Data are obtained as duplicates from at least three independent experiments and shown as mean ± SEM. P-value was determined using ANOVA with Tukey’s test for multiple comparisons. CTRL=Control group (Stimulation with phosphate-buffered saline (PBS)); ISO=Isoprenaline stimulation group; CGP = β1-adrenoceptor inhibitor; ICI = β2-adrenoceptor inhibitor; mRNA=messenger ribonucleic acid; ARG1 = Arginase 1; CTGF=Connective tissue growth factor; CXCL10 = C-X-C motif chemokine 10; CD=cluster of differentiation.

To simulate the SNS and assess its effect on macrophage phenotype, PMA-differentiated THP-1 cells were stimulated with β1-adrenoceptor agonist ISO or PBS (CTRL), with or without β1-adrenoceptor antagonist CGP or β2-adrenoceptor antagonist ICI (Fig. 5a). In the ISO group ARG1 and CTGF expression was 570.4 ± 88.4% (*p* < 0.001 vs. CTRL) and 1061.0 ± 230.4% (*p* < 0.001) higher than in CTRL, respectively. When compared to the ISO group, ARG1 and CTGF expression were significantly smaller in CGP (ARG1 *p* < 0.001 vs. ISO, CTGF *p* < 0.001) but not ICI (ARG1 *p* = 0.990 vs. ISO, CTGF *p* = 0.723), suggesting mediation via β1-adrenoceptor (Fig. 5b). In contrast, gene expression of CXCL10 and CD80, which are upregulated in proinflammatory M1 macrophages, were 33 ± 5.5% (*p* = 0.010 vs. CTRL) and 30.6 ± 6.0% (*p* = 0.004) smaller in the ISO group compared to CTRL. In the β1-blockade group (CGP + ISO, CXCL10 *p* < 0.001 vs. ISO, CD80 *p* = 0.002) but not β2-blockade group (ICI + ISO, CXCL10 *p* = 0.999 vs. ISO, CD80 *p* = 0.727, Fig. 5c) CXCL10 and CD80 expression were significantly higher compared to ISO. The expression of β1- and β2-adrenoceptors in PMA-differentiated THP-1 cells was confirmed by western blot analysis (Supplemental material online).

## Discussion

In rats undergoing experimental MI, RDN prevented LV systolic dysfunction, as assessed by cardiac MRI. RDN + MI rats exhibited smaller scar sizes and less LV fibrosis compared to MI rats, which was associated with reduced CD206 expression. Mechanistically, our findings suggests that β-adrenergic stimulation of PMA-differentiated THP-1 cells induces a shift towards a profibrotic macrophage phenotype, which could be reversed by β1-adrenoceptor blockade. Collectively, this study demonstrates that inhibiting sympathetic nerve activity trough RDN mitigates systolic LV dysfunction post-MI which is associated with reduced myocardial fibrosis and CD206 expression.

MI is a leading cause of morbidity and mortality worldwide. Its management is time-sensitive, as cardiomyocyte loss due to ischemia results in the formation of fibrotic scar, often progressing to ischemic HF. The subsequent remodeling of the remaining myocardium further impairs cardiac function^26^. The herein presented transcriptomic analysis provides an unbiased assessment of the myocardial response and reveals that RDN suppresses fibrosis and inflammation related pathways at the whole transcriptome level, with clear separation of CTRL, MI, and RDN + MI samples by PCA. To our knowledge, this represents the first high-throughput transcriptomic profiling of myocardial tissue in the setting of RDN in experimental MI, providing an unbiased systems-level view that complements our functional and histological findings. Quantifications were performed 4 weeks post MI and therefore reflect the mature scar and remodeling outcome, rather than the initial extent of necrosis shortly after MI. The smaller chronic scars in the RDN + MI group are result of differences in infarct expansion, extracellular matrix deposition and scar maturation as well as inflammatory cell dynamics. Future studies with early infarct assessment will be required to determine whether RDN affects initial injury as these measurements at an early time point after MI were not conducted in this study.

Ischemic HF is often complicated by sympathetic overactivity^5^, which was also observed in our study, where animals with MI exhibited significantly elevated renal NE levels compared to CTRL. RDN modulates efferent and afferent sympathetic signaling, effectively reducing SNS activity. In humans, this catheter-based procedure is safe and recommended by current guidelines for patients with uncontrolled resistant hypertension or inability to tolerate antihypertensive drugs^27,28^. Importantly, preclinical studies have shown various cardioprotective effects of RDN beyond blood pressure lowering^23,29–32^, suggesting that attenuating sympathetic activity via RDN could offer a novel therapeutic strategy for MI and concomitant LV remodeling. Renal TH and NE levels were significantly smaller in RDN + MI rats compared to CTRL rats, confirming the sympatholytic effects of RDN^16,22,25,29^ in our animal model. RDN did not affect kidney function or other laboratory parameters. Cardiac MRI demonstrated higher LVEF in RDN + MI compared to MI rats, with a nearly 50% reduction of myocardial BNP levels in the RDN + MI group, further supporting the hypothesis that RDN mitigates the development of systolic LV dysfunction. This finding is in line with previous studies showing improved cardiac function following surgical and radiofrequency RDN^15–18,22^.

Elevated levels of bone marrow NE and TH after MI promote mobilization of innate immune cells^2,10^. Interestingly, SNS activity, as measured by norepinephrine (NE) levels, correlates with myeloid cell numbers in both patients and animals models of MI^22^. RDN reduces NE levels in the spleen after MI and attenuates the expression of adhesion receptors on splenic myeloid cells, which reduced their migration to the heart^33^. Catecholamines, by both sympathetic nerve fibers and leukocytes, promote macrophage progenitor cell proliferation and differentiation^8^. In vivo polarization is complex and cannot be fully explained by a simple dichotomous model^34^, however, transcriptomic analyses show that macrophages shift from a pro-inflammatory, matrix-degrading phenotype early post-MI to a reparative, fibrogenic phenotype by day 7 in C57BL/6J male mice^14^. CD206, a marker for profibrotic macrophages, is predominantly expressed in this subtype^35^. CD206 expressing macrophages have been considered beneficial during the acute and subacute phase of MI. Mice lacking CD206 expressing macrophages at day 7 post-MI showed poor survival and frequent cardiac rupture due to reduced collagen content^36^. However, remodeling in the more chronic phase post-MI represents a different entity, where the relevance of reparative macrophages is less clear. In our study, a dramatic increase in myocardial collagen content and fibrogenic markers was detected 4 weeks after MI, which was significantly smaller in RDN-treated rats. Fibrotic remodeling was correlated with CD206 expression, and less CD206 expression was present in RDN-treated rats. These findings provide a potential explanation for the anti-fibrotic effects of RDN, consistent with previous studies^17,29,37^. Targeting of CD206 has shown benefits in chronic remodeling, such as in interstitial lung fibrosis^38,39^. At that timepoint as late as 4 weeks after MI, no significant cytokine alterations through RDN were detected (Supplement material online).

RDN reduces efferent sympathetic outflow to organs beyond the kidneys^40–42^, raising the question of whether RDN affects sympathetic innervation of bone marrow or spleen. Alterations in the sympathetic innervation of these organs could be linked to the observed changes in macrophage phenotype. However, investigating this was beyond the scope of our study, and further research is needed to explore the effects of RDN on bone marrow and splenic innervation and its subsequent impact on immune cell populations.

To assess the role of sympathetic overactivity on macrophage phenotype in vitro, PMA-differentiated THP-1 cells were incubated with β-adrenoceptor agonist ISO and respective antagonists. These cells express β-adrenergic receptors with high affinity for ISO^43^. We confirmed β-adrenergic receptor expression in macrophages (see supplemental material) and observed a significant upregulation of profibrotic macrophage genes following ISO but not with concomitant ISO and β1-adrenoceptor antagonist stimulation. These results are conclusive, but they may not entirely reflect stimuli under in vivo conditions, because the major transmitter NE not only acts on β- but also on α-adrenoceptors. However, we did not observe significant regulation of above-mentioned genes in THP-1 cells upon norepinephrine stimulation (Supplement material online). The results could be an explanation for attenuated myocardial remodeling and fibrosis under β1-blockade^44,45^. These findings suggest that sympathetic overdrive promotes adverse myocardial remodeling by inducing a profibrotic macrophage phenotype, which can be reversed by RDN in vivo and β1-blockade in vitro. Further research is required to elucidate the molecular mechanisms underlying macrophage polarization due to sympathetic overactivity.

This study has four primary limitations. First, the sample size was relatively small, which may have reduced statistical power. Second, we did not collect blood samples during the acute post-MI phase, preventing us from assessing the impact of RDN on early immune cell or cytokine alterations. Although we did not assess cytokine or growth factor expression profiles of cardiac macrophages by flow cytometry, gene, protein and immunofluorescence analyses provide robust evidence that CD206-expressing macrophages are elevated 4 weeks post-MI and but not in RDN-pretreated rats. However, the connection between myocardial fibrosis and macrophage CD206 expression remains associative and further research upon macrophage phenotyping in sympatholytic therapies is ongoing. Third, to mechanistically test whether sympathetic activity contributes to early post-MI remodeling, RDN was performed 2 days before LAD ligation, a point in time at which denervation effects were probably already present^46,47^. While this timing supports mechanistic inference, the therapeutic efficacy of post-MI RDN will require dedicated follow-up studies. Fourth, the overall size of the MI scars in our rats is relatively small when compared to similar models described in the literature^48,49^. This may be attributed to several factors such as different methods for scar size estimation, differences in slices thickness and staining methods, and different post-infarction periods. Also, the surgeries performed shortly prior to the LAD ligation may have triggered systemic remodeling processes that could have influenced the subsequent myocardial infarction scar formation.

Taken together, RDN significantly attenuated LV dysfunction in rats with experimental MI. Mechanistically, we observed less myocardial fibrosis and smaller scar sizes, accompanied by a decrease in macrophage CD206 expression in RDN-treated rats.

## Supplementary Information

Below is the link to the electronic supplementary material.

Supplementary Material 1

## Data Availability

All data generated or analyzed during this study are included in this published article (and its Supplementary Information files).
