# Supplementary material for: Renal denervation attenuates cardiac fibrosis and improves left ventricular function in rats with myocardial infarction
Source: Sci Rep. 2026 Apr 26;16:13416. doi: 10.1038/s41598-026-50195-w (PMC13111652; doi:10.1038/s41598-026-50195-w)
Supplement: Supplementary file 1 — Supplementary Material 1 [file 41598_2026_50195_MOESM1_ESM.pdf]

Online Supplement

Materials

Buffers

TRIS-EDTA buffer

5.0 mmol/L Tris(hydroxymethyl)aminomethane, 2.0 mmol/L Na-EDTA, pH 7.4

Phosphate-buffered Saline

137 mmol/L NaCl; 2.7 mmol/L KCl; 4.3 mmol/L Na_2HPO_4; 1.47 mmol/L KH_2PO_4, pH 7.4

Homogenization buffer for Western Blot

5 mmol/L EDTA; 25 mmol/L NaF; 300 mmol/L sucrose; 30 mmol/L KH_2PO_4, pH=7.0

TaqMan Probes

Probe	Species	Assay ID
GAPDH	Rat	Rn01775763_g1
BNP	Rat	Rn00580641_m1
COL1A	Rat	Rn01463848_m1
TGFb	Rat	Rn00572010_m1
CTGF	Rat	Rn01537279_g1
CD68	Rat	Rn01495634_g1
CD206	Rat	Rn01487342_m1
CXCL10	Rat	Rn01413889_g1
GAPDH	Human	Hs02758991_g1
ARG1	Human	Hs00163660_m1
CTGF	Human	Hs01026927_g1
CXCL10	Human	Hs00171042_m1
CD80	Human	Hs01045161_m1

Antibodies

Antibody	Species	Dilution	Manufacturer / ID
Anti-GAPDH	Mouse	1:15000	Merck MAB 374
Anti-Tyrosine hydroxylase	Rabbit	1:1000	Abcam Ab112

Anti-CTGF	Rabbit	1:1000	Abcam Ab227180
Anti-Mannose-Receptor	Rabbit	1:1000 (WB) / 1:50	Abcam Ab64693
Anti-mouse IgG-HRP	Goat	1:10000	Bio-Rad 1706516
Anti-rabbit IgG-HRP	Goat	1:10000	Bio-Rad 1721019
TRITC Anti-Rabbit	Donkey	1:100	Jackson IR 111-025-144

Supplemental Figures

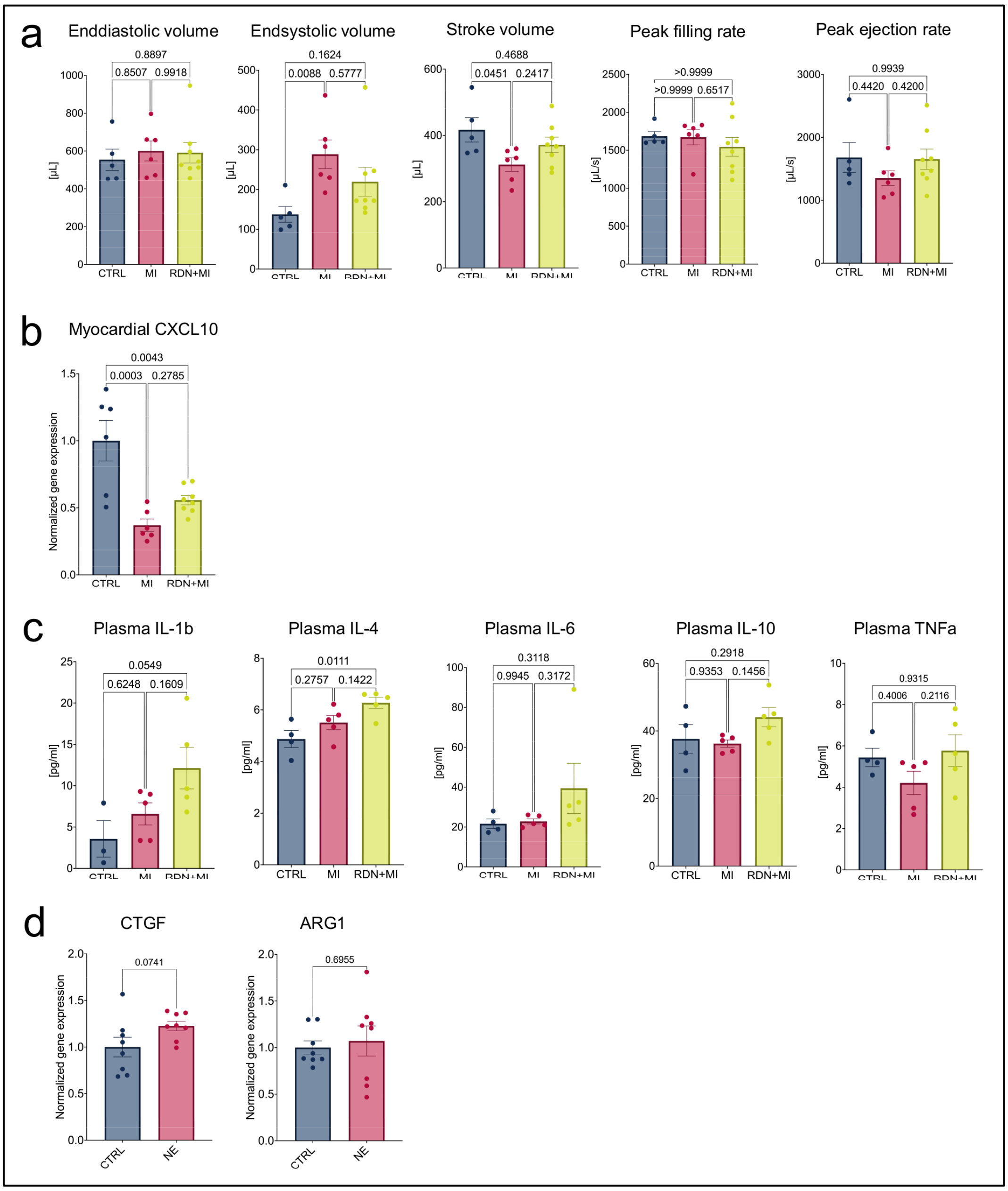

Supplemental Figure 1

(a) Quantification of additional cardiac volumes and filling rates as assessed by MRI. MI rats (n=6) displayed elevated end systolic volumes and reduced stroke volumes when compared with CTRL (n=5), which was not significantly altered in RDN+MI (n=8). (b) Gene expression analysis revealed significant attenuation of myocardial CXCL10 in MI (n=6) and RDN+MI (n=8) compared to CTRL (n=6). (c) Multiplex cytokine analysis of rat sera revealed no significant differences between the three groups. Data are shown as mean±SEM. P-value was determined using ANOVA with Tukey's test for multiple comparisons or using Kruskal-Wallis test if normality could not be assumed.

CTRL=Control group (Sham RDN-Sham MI); MI=Myocardial infarction group (Sham RDN-MI); RDN+MI=Renal Denervation and myocardial infarction group (RDN-MI); MRI=Magnetic resonance imaging; CXCL10=C-X-C motif chemokine 10; IL=Interleukin; TNFa=Tumor necrosis factor alpha.

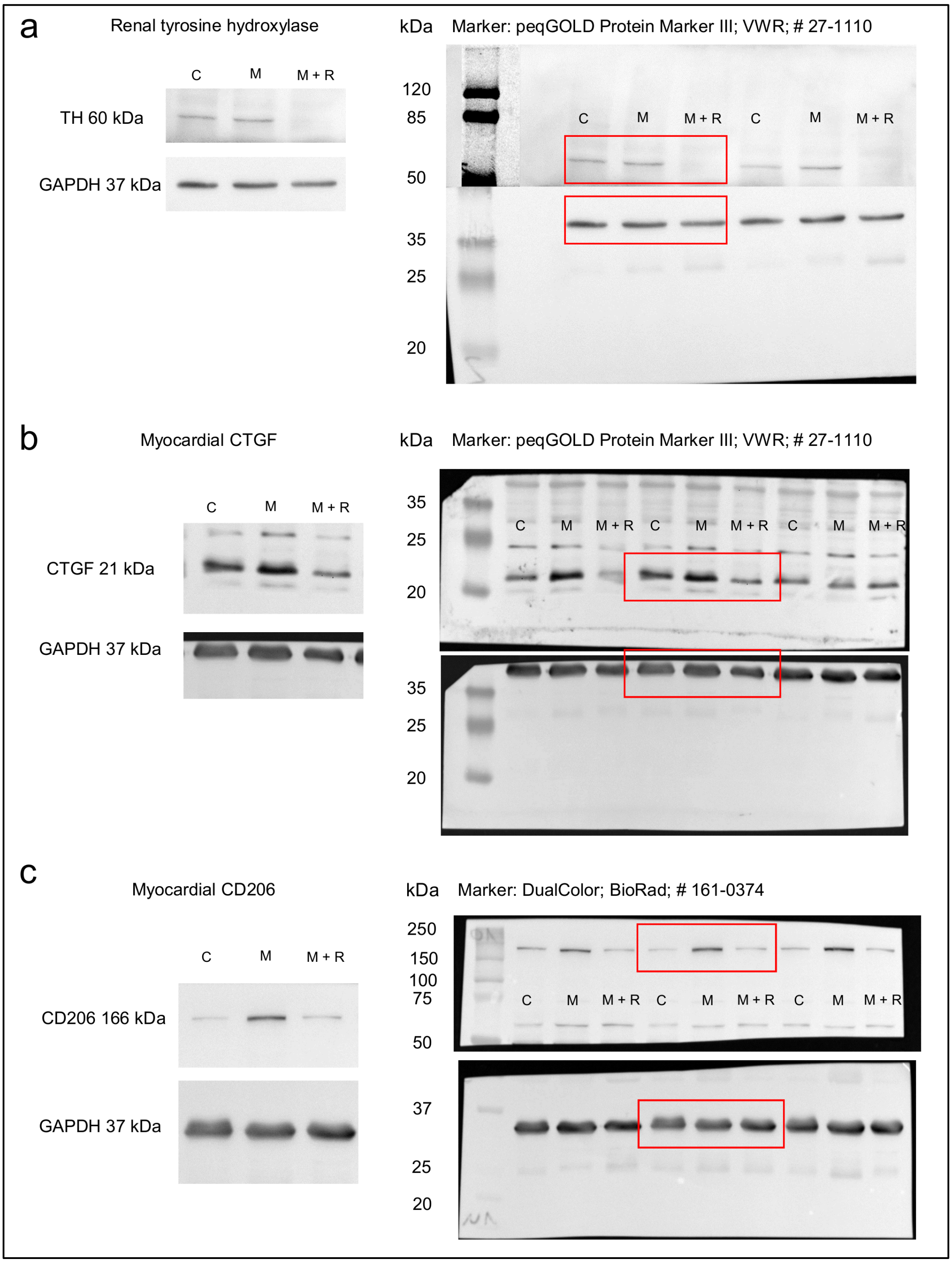

Supplemental Figure 2

(a) Original gel of renal tyrosine hydroxylase. (b) Original gel of myocardial CTGF. (a) Original gel of myocardial CD206. C=Control group (Sham RDN-Sham MI); M=Myocardial infarction group (Sham RDN-MI); R+M=Renal Denervation and myocardial infarction group.
